# Synthesis and Photolysis Properties of a New Chloroquine Photoaffinity Probe

**DOI:** 10.3390/molecules29051084

**Published:** 2024-02-29

**Authors:** Benita Kapuku, D. Scott Bohle

**Affiliations:** Department of Chemistry, McGill University, 801 Sherbrooke St. W., Montreal, QC H3A 0B8, Canada; benita.kapuku@mail.mcgill.ca

**Keywords:** antimalarial, drug target, photoaffinity label

## Abstract

A new chloroquine-derived photoaffinity probe has been prepared by a convergent synthesis from derivative of 4,7-dichloroquinoline and N1,N1-diethyl-N4-methylpentane. The features of this probe are a unique 3-azido photolabel, the pyridine ring of the quinoline, and the presence of a secondary amine at the 4-position of the quinoline. These features, particularly the 4-amino methylation, prevent triazole formation through combination of the 3-azide and the 4-amine. This undergoes facile cleavage with exposure to a medium-pressure mercury lamp with a 254 nm excitation wavelength. Trapping of the nitrene byproduct is accomplished with its reaction with N-phenylmaleimide as its cycloazidation product. The structure of a ring-opened DBU amine has been structurally characterized.

## 1. Introduction

Quinoline-based antimalarials have been in use for centuries; quinine, the godfather of antimalarials, has been in use since the 1600s in Europe. Quinine was first isolated in 1820, mepacrine was first synthesized in 1930, chloroquine was first synthesized in 1934, and mefloquine was developed in the 1970s. Despite their long tenure on the commercial market, their mechanism of action is still a matter of great debate. There is a consensus that blood-stage aminoquinolines interact with heme to inhibit the proliferation of the parasite; however, it is not believed to be their sole function. The mechanism of quinoline accumulation into the active site and its targets are still being investigated.

In an attempt to determine the proteins with which the antimalarial drugs interact, several photoaffinity labels have been synthesized. These labels are convenient because they form strong covalent bonds with the surrounding proteins, thus allowing for the isolation and identification of the ligated product.

**Figure 1:**
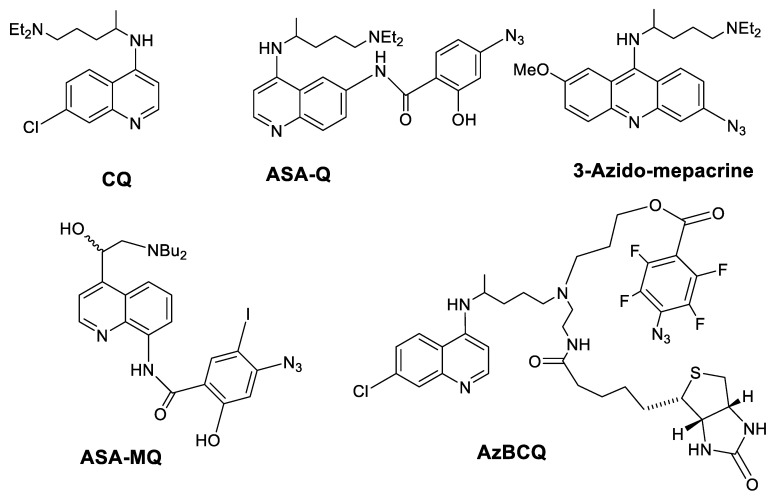
Known quinoline antimalarial photoaffinity labels.

Although the focus of considerable sustained effort, there remain many gaps in our understanding of the mechanism of quinoline antimalarial activity. The exact mechanisms of its efflux, importation, and substrate binding all need further clarification, particularly on the molecular level. Potentially useful in this regard are photoaffinity labels, with the known labels for studying quinoline antimalarial bioactivity shown in Figure 1. These include azide species and biotinylated derivative labels such as N-(l-(l-diethylamino-l-methylbutylamino)quinolin-6-yl)-4-azido-2-hydroxybenzamide (ASA-Q) [1], 3-azido-9-[[4-(diethylamino)-1-methylbutyl]amino]-7-methoxyacridine (3-azido-mepacrine) [2], N-[4-[I-hydroxy-2-(dibutylamino) ethyl] quinolin-8-yl]-4-azido-2-hydroxybenzamide (ASA-MQ) [3], and perfluorophenylazido biotinylated chloroquine (AzBCQ) [4]. By definition, there is no completely satisfactory photoaffinity probe; the incorporation of a photolabile group invariably causes a change in structure. However, the prior probes have other drawbacks: ASA-Q completely removes the 7-chloro group, which is known to be essential for the inhibition of hemozoin formation and introduces an amide, hydroxy, and phenyl group, which can form hydrogen bonds and modulate π-stacking interactions. These are unfavorable modifications as they can interact with the surrounding environment. 3-azido-mepacrine replaces the 7-chloro group for the azide; this is unfavorable because as mentioned above, the chloro group is required for hemozoin inhibition. ASA-MQ, which is a photoaffinity label of mefloquine, does a poor job of retaining the core functions of the parent molecule. Desneves et al. claim that ASA-MQ is 10-fold more potent than mefloquine and that ASA-MQ is inactive in the K1 Mef2 strain, which is selected in vitro for resistance to mefloquine. However, there is no mention of any possible cross-resistance evident for the strain. AzBCQ is a chloroquine photoaffinity label that contains a biotin tag and a perfluorophenylazido group; the parent molecule remains unmodified, as all essential features are retained, but there is the addition of large groups on the side chain.

**Figure 2:**
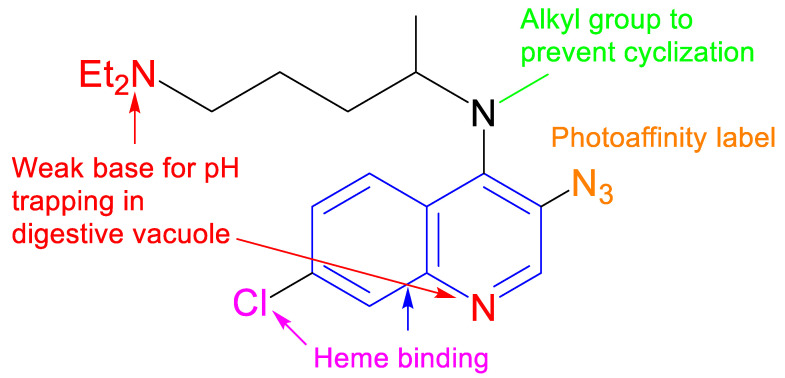
Structure of novel 3-azidomethylchloroquine, in which all important chloroquine features are present.

In this contribution, we will describe in detail the synthesis of the photoaffinity label, Figure 2, which contains minimal modifications of the parent molecule, ones in which all essential features of chloroquine activity remain unmodified. The photolytic and photophysical properties have been determined as the optimal wavelength and conditions for their activation. 

## 2. Results and Discussion

**Scheme 1:**
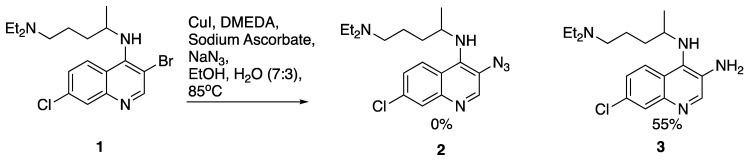
Ullman amination of aryl bromide in 30% aqueous solution in the presence of a ligand and reducing agent.

**Scheme 2:**
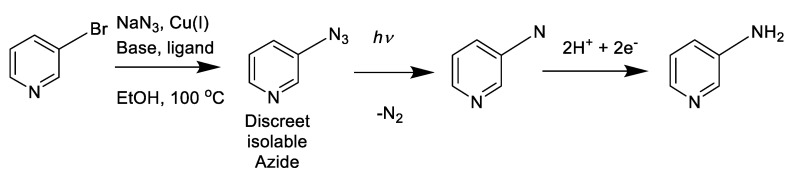
Proposed mechanism for aryl amine formation.

We synthesized a chloroquine photoaffinity labelled analog with minimal modification of the quinoline structure. The aryl azide was placed at the 3-position, a position that our group has previously taken advantage of [5,6]. The strategy for this preparation is to use an Ullman coupling for the formation of a C_sp2_—heteroatom bond at the 3-position [7]. Typically, this is carried out under aerobic conditions with a copper catalyst and ligand in a polar solvent, with or without the presence of a base [8,9,10]. In our experimentation, the azidation reaction from an aryl bromide did not yield any trace of aryl azide. Rather, an aryl amine was the only product observed (Scheme 1). A further search of the literature shows that direct amination of aryl bromide with sodium azide as an amine source is a common observation [11,12,13]. Thatcher et al. explained this to be a result of thermal decomposition due to the instability of the aryl azide (Scheme 2) [14,15,16]. Similarly, Alami et al. proposed a mechanism of amine formation via a nitrene intermediate [12]. Furthermore, they were unable to gather experimental evidence of azide formation, but believe that the aryl azide is first formed and through thermo-initiation, N_2_ is liberated to form a nitrene, which abstracts protons and electrons from the solvent.

However, Helquist et al. later disproved this by heating an aryl azide in DMSO for 72 h, and they found that the azide remained intact [11]. They then heated an aryl bromide in the presence of a copper catalyst and dimethylethylenediamine (DMEDA) and only recovered an azo compound. They determined that amine formation from an azide precursor is dependent on the presence of a copper catalyst and excess sodium azide. In our experiments, we did not isolate the desired azido product or any azo product; only aryl amine was detected and isolated. As expected from prior work, regardless of the reaction conditions, the aryl amine is the only isolated product. 

**Scheme 3:**
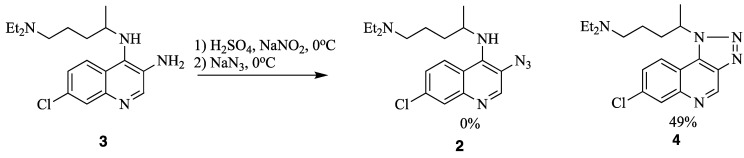
Azidation of aryl amine leads to the formation of a triazole chloroquine product.

With the aryl amine in hand, we proceeded to form the aryl azide via a route that first involves the formation of a diazonium salt with the use of sodium nitrite in the presence of sulfuric acid. Following the formation of the diazonium intermediate, sodium azide was added to the reaction mixture to supposedly form the aryl azide. However, the aryl azide was never isolated; rather, a cyclized triazole compound was isolated (Scheme 3). This structure was confirmed by NMR and HRMS spectroscopies. Formation of the triazole indicates reactivity at the 4-position of the secondary amine. In order to prevent cyclization, the reactive amine had to be permanently blocked because if we were to temporarily protect the secondary amine and then deprotect after azide installation, then there would be a high probability of cyclization still occurring. However, this method was low yielding and, in some instances, resulted in a dimethylated product (Table 1). Dimethylation occurs at the tertiary and secondary amine.

Varying reaction conditions did not improve product yield or selectivity, and the HRMS continuously showed the formation of mono and dimethylated chloroquine. The tertiary alkyl amine had a pK_a_ of 10.1, which explained the difficulty in producing a regioselective product. We then attempted methylating the secondary amine via the formation of formamide followed by its reduction to produce the desired methyl chloroquine (MeCQ).

Formic acid, formamide, and sodium ethoxide were used as formylating agents; however, these reactions did not yield the desired result [17,18,19,20]. When the reaction was monitored for over 24 h, only unreacted starting material was present (Table 2). Following the mentioned failed attempts, we deduced that there was too much steric hindrance at the reaction center due to the flexibility of the alkyl side chain and quinoline ring.

It was decided that methylation of the side chain followed by an S_N_Ar reaction would be the most efficient method for the synthesis of MeCQ (Table 3). Formylation of the primary amine with formic acid and acetic anhydride proved to be unsuccessful; this was probably due to the insufficient formation of acetic formic anhydride before the addition of primary amine. However, the reaction with primary amine and formic acid produced the desired formamide in moderate yield [17]. Reactivity was improved using ethyl formate to yield 97% of a product and easy recovery with a simple acid and base workup [21]. When ethyl formate was used, the side chain did not become protonated, as with formic acid, and the primary amine was more nucleophilic, resulting in our higher yield.

Once the formamide is formed, the final step is its reduction with lithium aluminum hydride to give the desired methylated side chain in moderate yield [22].

Lastly, in order to form the sought MeCQ, we performed a condensation reaction between 4,7-dichloroquinoline (DCQ) and the alkyl diamine. Generally, this type of condensation reaction happens under basic conditions. As the alkyl side chain contains a basic secondary amine, we opted to carry out the reaction with the amine acting as both the substrate and the base, and in some examples as the base and solvent (Table 4).

**Scheme 4:**
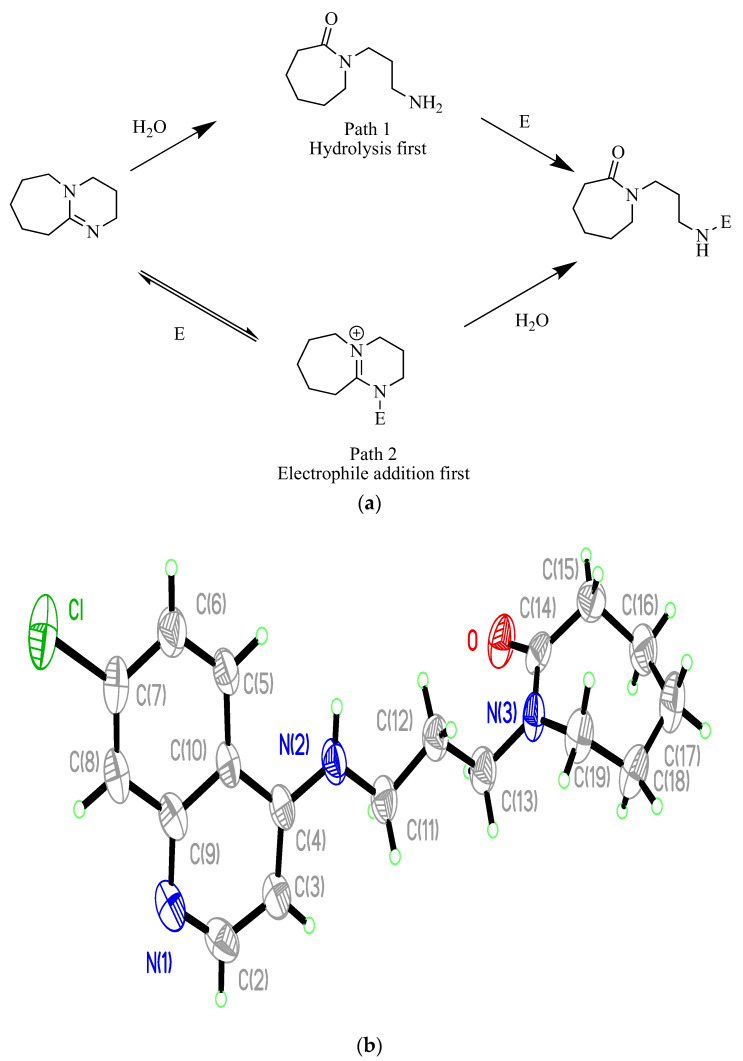
(**a**) Potential pathways for DBU reaction with electrophile. (**b**) Thermal ellipsoid plot of 7-chloroquinoline adduct of DBU. See the experimental section for further details.

In prior reports, the use of thermal activation for this type of condensation reaction with primary amines gives moderate to excellent yields [23,24,25]. However, in our experiments the use of the diamine as a base resulted in a low yield of product, even at elevated temperatures. The use of a polar solvent such as ethylene glycol did not improve the yield. It became clear that the diamine was not nucleophilic enough to initiate the S_N_Ar reaction; thus, we used stronger bases such as triethyl amine (NEt_3_), 1,8-diazabicyclo[5.4.0]undec-7-ene (DBU), N,N-diisopropylethylamine (DIPEA), and potassium tert-butoxide (^t^BuOK). NEt_3_ did not result in product formation, nor did DIPEA with various polar solvents. DBU gave 10% product yield at 80 °C; however, when the temperature was increased to 125 °C, the product contained 82% of a ring-opened DBU adduct (Scheme 4). Such adducts of ring-opened amidine bases have been observed in the literature whilst in the presence of an inorganic base [26,27]. A recent publication from Merck scientists details their discovery of a ring-opened DBU adduct during the process development of a hepatitis C drug [28]. Hyde et al. hypothesized that the hydrolysis of DBU occurs in trace amounts of water and high temperatures or during an aqueous workup When examining the reactivity of electrophilic reactions in the presence of DBU, they proposed two pathways to the observed ring-opened DBU adduct. In the first pathway, the amidine base is hydrolyzed, generating a primary amine that reacts with an electrophile. In the second pathway, a cationic intermediate is formed by way of a reversible interaction of an amidine and an electrophile, which then reacts with trace amounts of water or an aqueous workup that results in the ring-opened product. In our experiments, whilst monitoring the reaction with TLC, the product was formed before quenching the reaction; thus, the trace amounts of water found in the reagent bottle are responsible for the hydrolysis of DBU.

When potassium tert-butoxide was used, it led to the decomposition of the reaction mixture. The condensation catalyzed by palladium acetate was also unsuccessful.

**Scheme 5:**
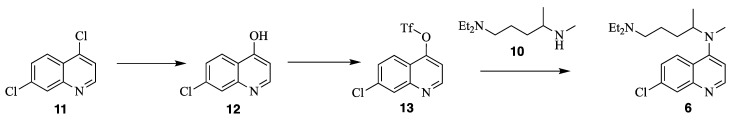
Increasing the electrophilicity of C-4 by transformation to a quinoline triflate.

We concluded that the C-4 was not sufficiently electrophilic for this addition and opted for a better leaving group than chloride. Triflic acid has a pK_a_ value of −14, whereas hydrochloric acid has a pK_a_ of −8; therefore, we opted for the weaker conjugate base, being the triflate. The installation of the triflate involves nucleophilic substitution with acetic acid to give 7-chloroquinolin-4-ol. With the hydroxy in hand, we then carried out a carbonyl electrophilic reaction to afford the triflate quinoline [29]. The final step to obtaining MeCQ was condensation with the methylated side chain (Scheme 5).

Initially, the reactions were placed in an 80 °C oil bath, and the reaction was monitored for one hour. When monitoring the TLC, if no product formed after one hour then the flask was transferred to a 125 °C oil bath and heated for a further hour before quenching. It is evident from the results (Table 5 and Table 6) that the use of a weaker base as a leaving group more than doubled the product yield, thus proving the hypothesis for a need to make C-4 more electrophilic. However, although the yields were improved, they were still poor.

We then decided to take the unconventional route for this type of condensation reaction, to carry out the reaction under acidic conditions. In the case of chloroquine and 4-aminoquinolines, the use of acidic conditions for this condensation reaction is common, and in some examples of condensation under basic conditions the yields are good, being thermally activated without the use of promoting Lewis acids. There are examples in the literature of this reaction being carried out in weakly acidic conditions with the use of phenol and in strongly acidic conditions with the use of hydrochloric acid [30,31,32,33]. The acid protonates the secondary amine, which forms a quaternary ammonium cation, which would be slightly less basic than triethylamine.

Phenol is a weak acid with a pK_a_ value of 10, and it did not improve the reaction yield compared to the use of strong bases. It is important to note the 7-chloro-4-phenoxyquinoline side product formation in the reaction mixture containing phenol and triethylamine. This side product has been reported by other research groups who did not use a base [34]. As the use of phenol did not optimize the reaction, a stronger organic acid, p-toluenesulfonic acid with a pK_a_ of −3, was employed. The reaction containing DMSO did not yield any product, which might be due to hydrogen bonding between the sulfonic acid and the sulfoxide preventing the protonation of the amine.

**Scheme 6:**
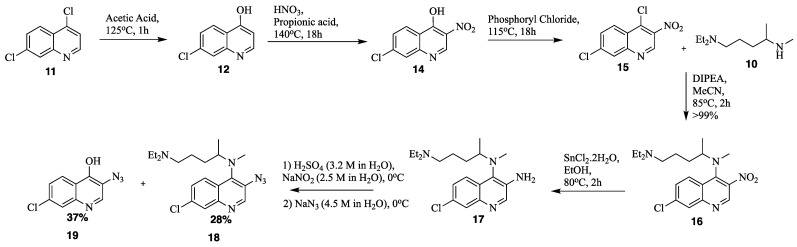
Linear synthesis of 3-azidomethylchloroquine.

Pan et al. reported similar reaction conditions between primary and secondary amines with 4-chloro-2-methylquinoline [35]. Under traditional heating conditions, a reaction mixture containing acid, a cyclic secondary amine, quinoline, and DMF at high temperatures gave moderate yields of ~50%. However, they determined that they could produce good to excellent yields under microwave-assisted conditions, and yields were improved when the solvent was removed from the reaction. In our experiments under a microwave-assisted system without the use of a solvent, we improved the yield of the reaction to 63%. With this improved result, we were satisfied with the yield and proceeded to the final stage of the synthesis to form 3-azidomethylchloroquine (3-N_3_MeCQ) (Scheme 6 below).

In prior work, we found that the highest-yielding route to 3-NH_2_CQ is via a series of S_N_Ar reactions to produce 4,7-dichloro-3-nitroquinoline (3-NO_2_DCQ).

**Scheme 7:**
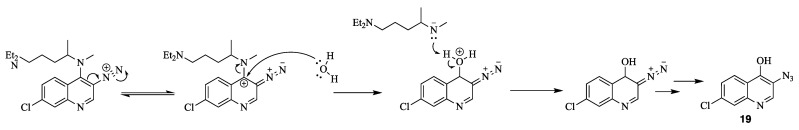
Proposed mechanism for 3-azido-7-chloroquinolin-4-ol formation.

Once 4,7-dichloro-3-nitroquinoline was synthesized, we carried out the condensation reaction to form the desired 3-NO_2_MeCQ precursor. Remarkably, in the case of 4,7-dichloro-3-nitroquinoline, the optimal reaction conditions involved the use of DIPEA in acetonitrile. This reaction occurs very quickly and gives quantitative yields. An explanation for this observation is the proximity of the electron-withdrawing nitro group, which greatly increases the electrophilicity of the C-4 position. The nitro group is then reduced with stannous chloride to afford 3-aminomethylchloroquine (3-NH_2_MeCQ). The final step is the conversion of the amine to azide via a diazonium salt with sodium nitrite and sulfuric acid. The diazotization reaction occurs immediately following the addition of sodium nitrite. However, a 3-azido-7-chloroquinolin-4-ol side product begins to form just as rapidly; thus, the addition of sodium azide followed by quenching the reaction with sodium carbonate must be performed within 10 min of starting the reaction. A proposed pathway for side product formation is shown below in Scheme 7. Once the diazonium ion forms, it can form resonance structures in which there is a cation of C-4; this electrophilic site can then be attacked by water. The quinoline-4-ol can then be deprotonated by the previous leaving group, a secondary amine, to form a 7-chloro-3-diazoquinolin-4-ol. In the last step of the synthesis when sodium azide is added, we achieve the formation of 3-azido-7-chloroquinolin-4-ol. The side product is insoluble in the acidic media and is filtered out of solution to allow the collection of the desired 3-N_3_MeCQ product.

In an attempt to reduce side product formation, we carried out the reaction in an organic solvent in order to minimize the water concentration. All reagents used have a degree of solubility in methanol, which was chosen as our solvent. The appropriate strong organic acid chosen was p-toluenesulfonic acid, and this reaction was carried out at 0 °C to room temperature and stirred for 18 h. TLC showed very little product conversion; when the time elapsed and purification was performed, only 1.4% of the product was isolated, with no formation of side product. Factors affecting product formation for the new method include the partial solubility of sodium nitrite and sodium azide, leading to a considerably lower rate of formation of the diazonium intermediate. Another factor affecting the formation of the product is the pH of the solution. In order for the nitrosonium ion to form, a very acidic environment is needed, which is not the case when p-toluenesulfuric acid is dissolved in methanol.

## 3. Photoaffinity Properties

With the desired 3-N_3_MeCQ in hand, we synthesized the first known quinoline-based photoaffinity label with the least modifications to the parent molecule. In doing so, we were able to retain all the important functionality for antimalarial activity.

**Figure 3:**
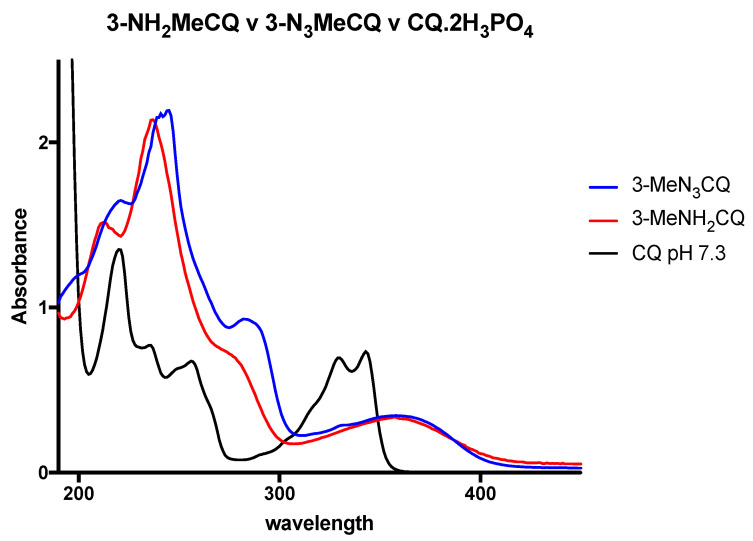
Overlaying spectra of 3-NH_2_MeCQ, 3-N_3_MeCQ, and CQ.2H_3_PO_4_ for comparison.

We first determined the UV–Vis spectroscopic difference between chloroquine, 3-NH_2_MeCQ, and 3-N_3_MeCQ. The spectroscopic profile of chloroquine was prepared in water and measured at pH 7.3, and the samples for 3-NH_2_MeCQ and 3-N_3_MeCQ were prepared in methanol, Figure 3. As expected, chloroquine has a very different profile to its derivatives, whereas there are several similarities between 3-NH_2_MeCQ and 3-N_3_MeCQ. As can be seen from the spectra, there is a 6 nm bathochromic shift from 237 nm to 243 nm in the λ_max_ of the amine compared to the azide. Both compounds have weak π → π* bands at 278 nm and 282 nm for the amine and azide, respectively, and weaker n → π* bands at 357 nm and 358 nm for the amine and azide, respectively. Chloroquine diphosphate is a colorless salt, which is proven in the spectra by all the absorbances being in the ultraviolet region. 3-NH_2_MeCQ and 3-N_3_MeCQ have a yellow tint, which is due to the tailed absorbance at 400 nm.

**Figure 4:**
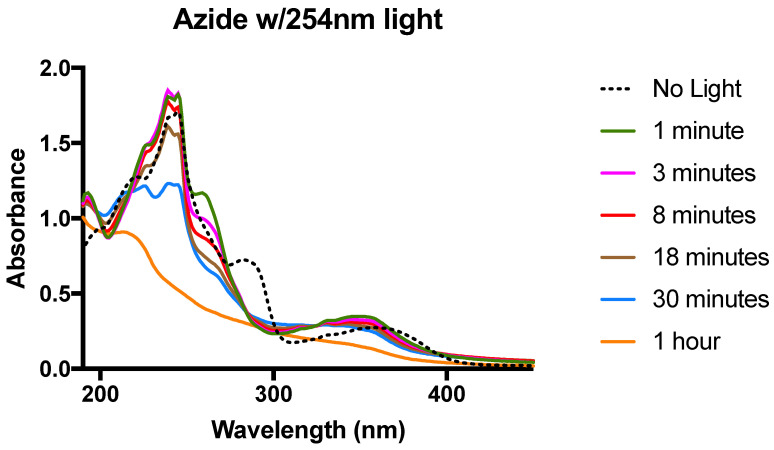
Photolysis of 3-N_3_MeCQ at 254 nm excitation.

We then determined the excitation wavelength for the photolabile moiety. It has been reported that aryl azides can be irradiated at 254 nm without causing damage to the molecule; therefore, we decided to test its stability at that excitation (Figure 4) [36,37]. The azide was first exposed to 254 nm using a pen light in the dark, exposure lasted 1 h, and the spectra were taken at different time intervals. As can be seen from the spectra, photoexcitation/decomposition is rapid as the π → π* band disappears within one minute. However, photodecomposition begins to take place within 30 min with the compound having little to no UV–Vis signature after one hour.

With this observation, we decided that photolysis at 254 nm was too energetic and tried photoexcitation at 365 nm. There are also many examples of azide irradiation above 300 nm that result in insertion reactions [1,38,39]. We carried out stability tests in water and methanol; the aryl azide remained intact after longer than 18 h with exposure to 365 nm of light.

**Figure 5:**
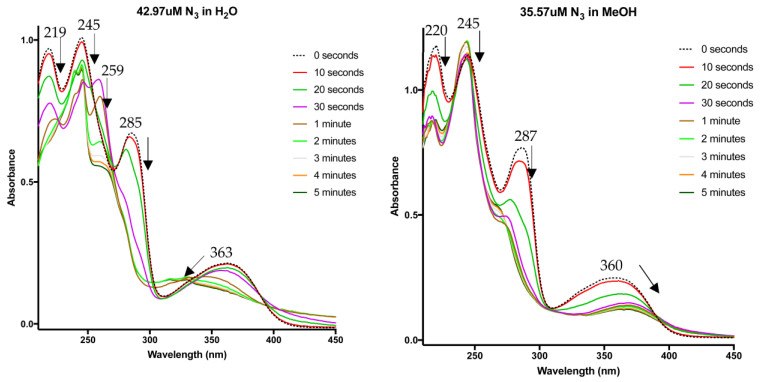
Photolysis of 42.97 μM N_3_ in H_2_O and 35.57 μM N_3_ in MeOH at 365 nm.

Due the high reactivity of the formed nitrenes, the observed spectra are the resulting product formation from the nitrene reacting with the polar solvent (Figure 5). These results show that the aryl azide is indeed photolabile; however, it is also a cause of concern that the reaction with the solvent will occur faster than insertion with a chosen molecule, making the detection of azide molecule insertion difficult with UV–Vis spectroscopy. Furthermore, binding regioselectivity is important, as the photolabel should ideally insert at the binding site.

**Figure 6:**
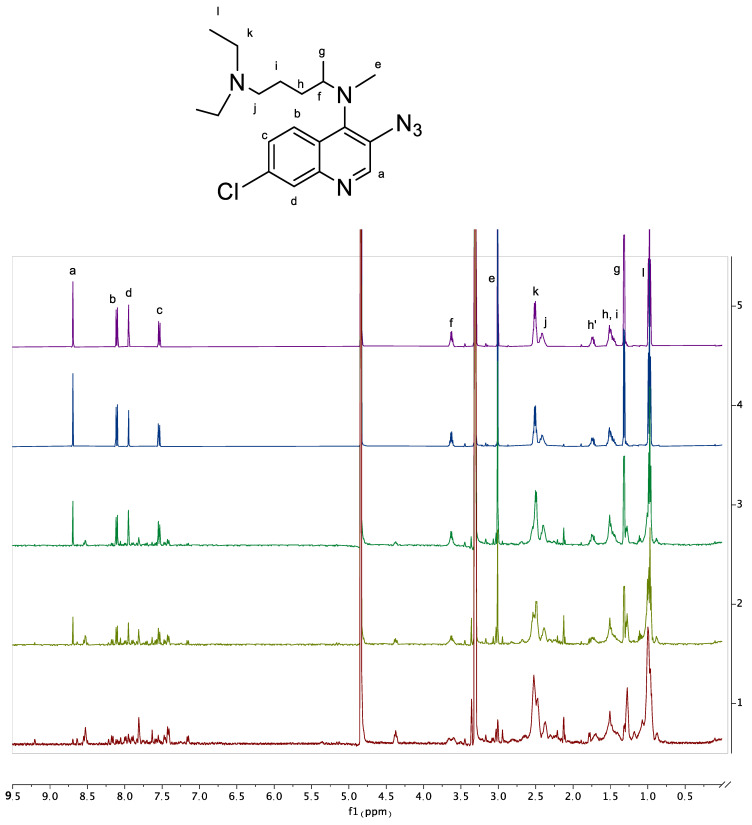
Photolysis of 3-N_3_MeCQ in d_4_- MeOD the lettering code are the assignments for the structure shown in the diagram.

We then carried out the same reaction in an NMR tube with a 365 nm pen light in the dark in deuterated methanol, Figure 6.

**Scheme 8:**
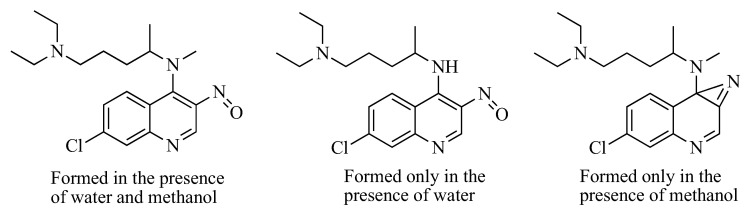
Proposed products for photoactivation products.

The irradiation of the aryl azide with 365 nm shows a clear change present in the structure. Changes become evident after 2 min of exposure. As seen in the UV spectra, the π → π* band disappears, and in the NMR spectra we can see the aromatic protons decrease in intensity as additional peaks arise. Additional photodecomposition occurs at longer exposure times, and the aromatic region is more affected than the aliphatic regions, as one would expect. Therefore, when carrying out photolysis experiments, exposure times did not exceed 10 min. High-resolution mass spectroscopy was carried out for the azide samples in water and methanol. We discovered the formation of three consistent molecular ions with their possible structures shown below. The formation of nitroso-methylCQ occurs in the presence of water and methanol when irradiated at 365 nm for 5 min. Interestingly, only in the presence of water do we achieve the formation of a nitroso-chloroquine derivatives that has lost a methyl group, Scheme 8. The mechanism for this loss is still unknown, but the exact molecular mass and chemical formula of the ion leads us to the believe that the shown structure is the most probable. Not so surprisingly, we have the formation of an intramolecular insertion product; however, it only formed in the presence of methanol. Formations of such azirines are well documented for phenyl azide, which exist in resonance with the corresponding ketenimine [40,41,42].

## 4. Nitrene Insertion

**Figure 7:**
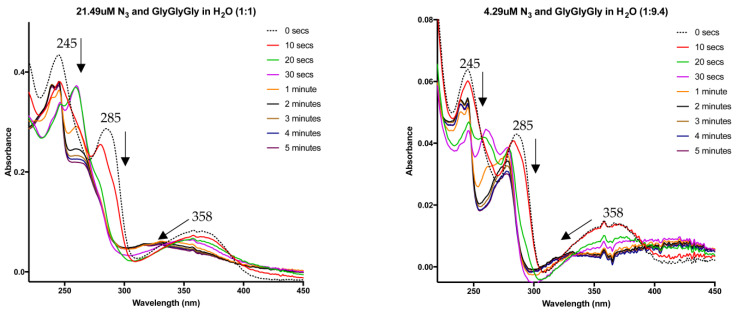
Photolysis of 21.49 μM 3-N_3_MeCQ with 22.32 μM GlyGlyGly and 4.29 μM 3-N_3_MeCQ with 40.18 μM GlyGlyGly at 365 nm excitation.

We carried out experiments to determine if the expected singlet nitrene intermediate that forms inserts into C-H, C-N, or C=C bonds. To achieve this, we first used a simple peptide GlyGlyGly. UV–Vis spectra of azide and GlyGlyGly were taken at different stoichiometries, and the change in the UV–Vis signature is evident at the different concentrations and times Figure 7. Furthermore, the equimolar mixture seems almost identical to that of 3-N_3_MeCQ in water alone. From the UV–Vis spectra, it is clear that there is a change in the aromatic system of the aryl azide when mixed with the peptide. To determine whether insertion occurred with the peptide, we carried out HRMS for the resulting product. However, there were no peaks related to an insertion product detected on the mass spec.

**Figure 8:**
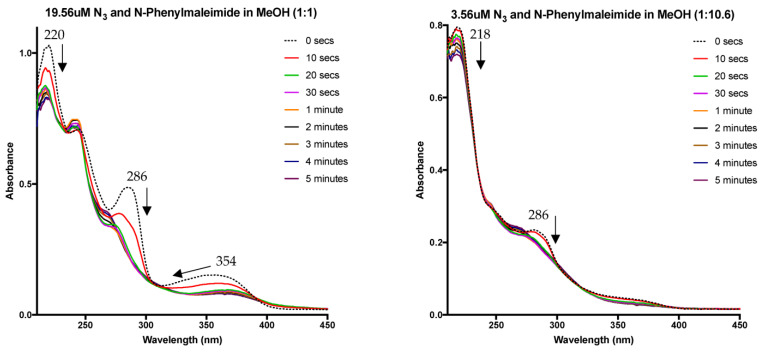
Photolysis of 19.56 μM 3-N_3_MeCQ with 18.77 μM maleimide, 7.11 μM 3-N_3_MeCQ with 33.37 μM maleimide, and 3.56 μM 3-N_3_MeCQ with 37.54 μM maleimide at 365 nm excitation.

We then decided to carry out UV–Vis and NMR studies to trap the nitrene with strong electron acceptors. First, we used N-phenylmaleimide with 3-N_3_MeCQ in the dark exposed to 365 nm of light. Similarly to GlyGlyGly, the equimolar sample had identical spectra to that of the azide in methanol alone. At higher concentrations, the absorbance associated with N-phenylmaleimide begins to obscure that of the quinoline; however, it seems to follow a similar pattern to that of the control experiment, Figure 8. The HRMS did not detect any molecular ions associated with a possible insertion product.

We then carried out the same experiment in an NMR tube to detect product formation at a higher concentration of both reagents.

**Figure 9:**
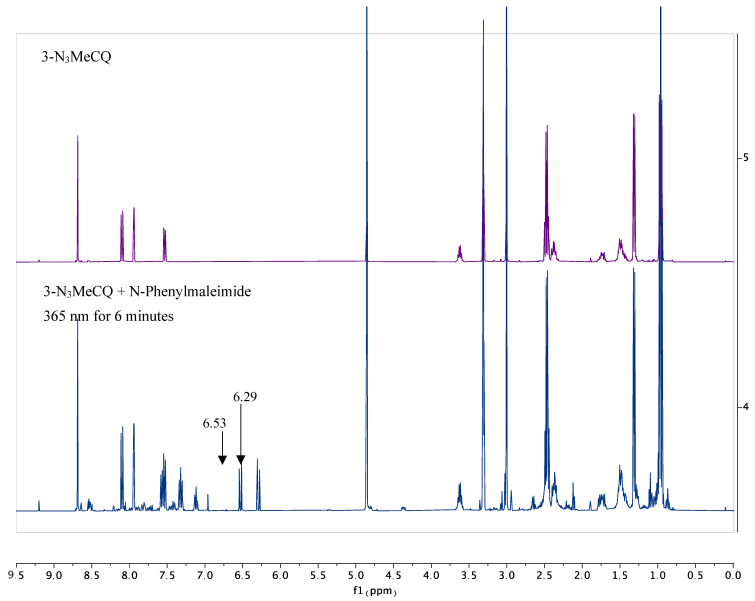
Photolysis of 26.7 mM 3-N_3_MeCQ and 26.6 mM phenylmaleimide at 365 nm in d_4_-MeOD.

**Figure 10:**
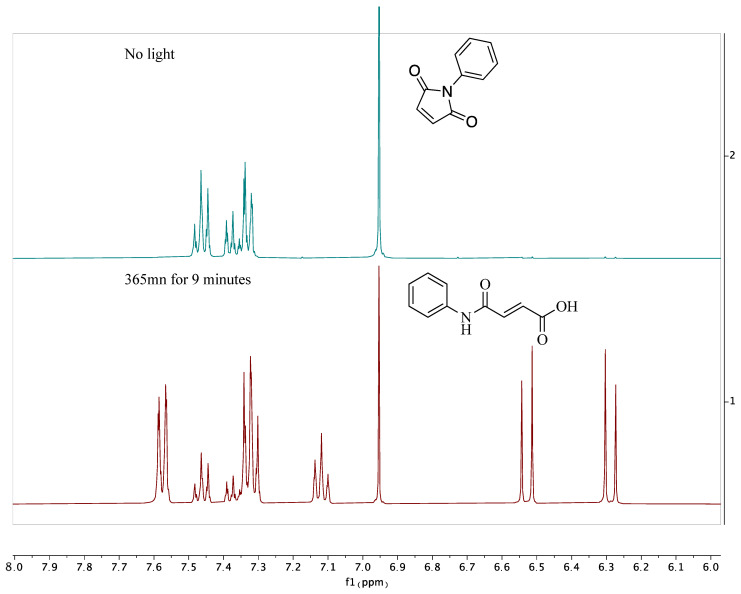
Comparison between activated and inactivated N-phenylmaleimide.

As shown in Figure 9 and Figure 10, doublets appear at 6.53 ppm and 6.29 ppm, which did not correspond to possible insertion products. To act as a control experiment, the substate N-phenylmaleimide in methanol was irradiated alone for a total of nine minutes, which saw the appearance of the vinyl protons, indicating independent activation of the maleimide. There is precedence for this in a paper by Hott and Heusinger published in 1977, in which they investigated the photolysis of maleimides in polar solutions by electron spin resonance [43]. They noted the detection of radical anions of maleimides in alcohol solutions. They determined that radical anions are the primary ion species as opposed to simple radicals, which would result in hydrogen atom radical adducts. They also noted the stability of radical anions in polar solvents, as they do not lead to ring opening. In our experiments, the radical anion is the predominate species at 58% compared to 42% of the unactivated maleimide. With the stoichiometry of 1:1 azide: maleimide, the radical anion appears to not be sensitive to the nitrene, as no insertion product could be detected by NMR.

We then decided to use a stronger electron acceptor tetracyanoethylene (TCNE). Murata et al. have reported that the photochemical reaction of mesityl azide with tetracyanoethylene with 360 nm ± 15 nm light produced two adducts via singlet nitrene trapping [44]. They also determined that the adduct did not form via a charge transfer complex after excitation of the charge transfer absorption band in acetonitrile with wavelength shorter than 520 nm for 20 h. They concluded that this was due to the instability of the complex.

**Figure 11:**
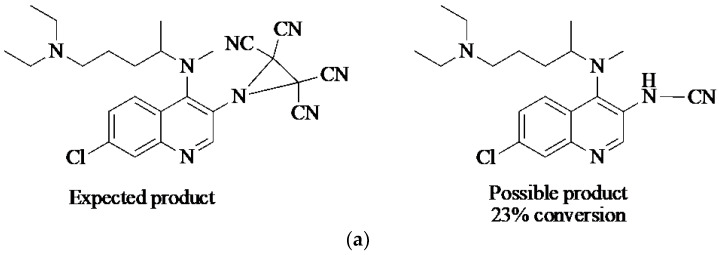

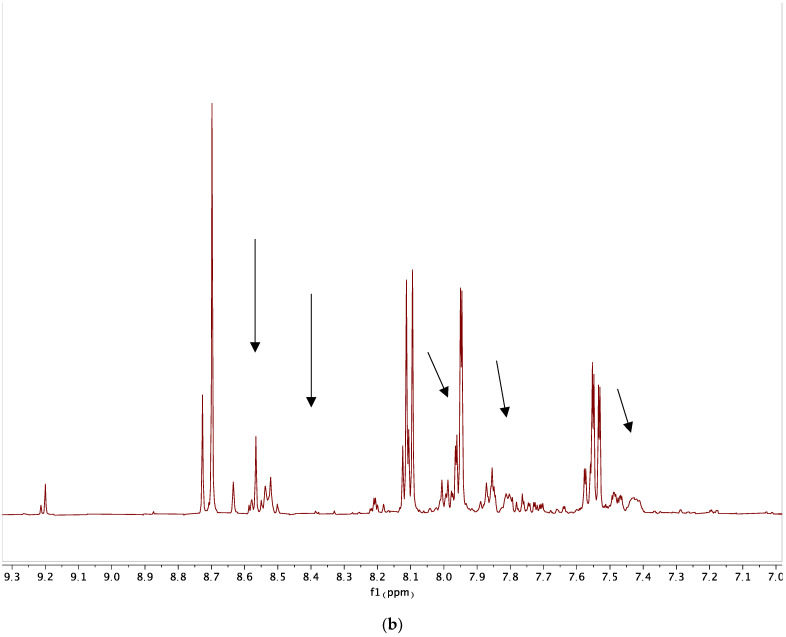
(**a**) Desired product vs. possible observed photolysis product; (**b**) NMR of photolysis product formation. Arrows indicate peaks believed to belong to the photolyzed product.

The irradiation of our aryl azide and TCNE for 9 min showed the formation of a minor product (23% conversion) in the aromatic region as well as a small downfield shift in the aliphatic protons, Figure 11. However, HRMS of the mixture did not detect the formation of the expected photolysis product.

The exact mass and chemical formula obtained from HRMS ([M + H^+^] *m*/*z*: 374.21) led us to the proposed structure of the reaction, where insertion occurs with the nitrile group rather than the expected alkene bond. Unfortunately, this product was not isolated, and longer excitation times led to the decomposition of the observed intermediary insertion compounds.

## 5. Conclusions

We have synthesized the first photolabile chloroquine derivative with minimal modifications of the parent molecule. The modification involves the installation of a methyl group on the 4-amino in order to prevent cyclization to a triazole derivative. In order to install the amine, we first had to methylate the side chain, followed by a condensation reaction with 4,7-dichloro-3-nitroquinoline. Once the desired azide was obtained, we caried out UV–Vis stability tests to determine the best suited excitation energy. We found that irradiation at 254 nm was too energetic and led to the decomposition of the aromatic system, and 365 nm of light was best suited as it activated the azide, causing a release of nitrogen to form a new compound. The reactive nitrene intermediate formed an adduct with the surrounding solvent, which was detected by high-resolution mass spectroscopy. We then carried out photolysis studies with GlyGlyGly, N-phenylmaleimide, and tetracyanoethylene, and discovered that the conditions for insertion are determined by the electron acceptor and the solvent. We were only able to detect an insertion product with tetracyanoethylene, in which the nitrene intermediate was inserted in a C-N triple bond.

More studies need to be carried out to determine the best conditions required for C-H, C-N, and C-C insertion as well as the optimal stoichiometry that will produce the highest conversion and yield. The results are very promising and indicate that this photolabeling agent could be used for mechanistic studies, and the developed synthesis for azide installation can be used for other 4-aminoquinolines.

## 6. Experimental

### 6.1. General Information

Glassware was taken directly from the oven (120 °C) and cooled in a desiccator before use. NMR experiments were carried out in Precision NMR sample tubes. All solvents and reagents were obtained commercially and used without further purification unless noted.

UV irradiation was carried out using Analytik Jena, Jena, Germany, UVP Pen-Ray UV lamp 254 nm and 365 nm. High-resolution mass spectroscopy (HRMS) was obtained by positive/negative ESI, or positive/negative APCI on a Bruker Maxis Impact QTOF, or a Thermo Scientific ExactivePlus Orbitrap, San Francisco, CA, USA, with results reported as mass/charge ratios (*m/z*). Thin-layer chromatography plates were visualized using ultraviolet light, 254 nm. Flash column chromatography was carried out manually using 230–400 mesh silica gel (Silicycle, Quebec, QC, Canada) and reagent grade solvents or by using Biotage Isolera One system with Biotage ZIP 30 g columns. Cole Parmer Microcomputer pH-Vision Model 05669-20 pH Meter, Walton, FL, USA was used with Sigma-Aldrich, Milwaukee, WI, USA micro pH combination electrode, glass body. The UV–Vis spectra were measured on Agilent 8453, San Jose, CA, USA.

^1^H and ^13^C NMR spectra were recorded at ambient temperature on Bruker AVIIIHD 400 MHz (^1^H 400 MHz, ^13^C 100 MHz) and Bruker AVIIIHD 500 MHz (^1^H 500 MHz, ^13^C 125 MHz) using tetramethyl silane as the internal standard. Chemical shifts are reported relative to the residual deuterated solvent peaks. Chemical shifts are expressed in parts per million (ppm = δ) values and coupling constants (J) in Hertz (Hz). The terms m, s, d, t, and q represent multiplicities of ^1^H NMR resonances: multiplet, singlet, doublet, triplet, and quartet, respectively. For previously unknown compounds, a combination of 2D experiments (COSY, HSQC, HMBC) were often used to complete assignment of ^1^H and ^13^C signals. ^1^H NMR signals are described by chemical shift δ (multiplicity, J (Hz), integration). ^13^C NMR signals are described by chemical shift δ and are singlets unless otherwise specified.

### 6.2. Experimental Procedure


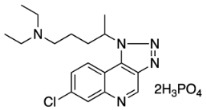
4-(7-chloro-1*H*-[1,2,3]triazolo[4,5-*c*]quinolin-1-yl)-*N*,*N*-diethylpentan-1-amine diphosphate (**4**)

7-chloro-*N*4-(5-(diethylamino)pentan-2-yl)quinoline-3,4-diamine (50 mg, 0.14 mmol) was dissolved in a solution of 3.2 M sulfuric acid (pH 0–1). This solution was cooled to 0 °C, and to it was added a 1 mL solution of 2.5 M sodium nitrite dropwise. The reaction mixture was stirred at this temperature for 15 min. After 15 min 1 mL 4.5 M sodium azide was added dropwise to the reaction mixture at 0 °C. Addition of azide resulted in visible N_2_ gas evolution. The reaction mixture was left to stir for a further 1 h and the mixture was allowed to warm from 0 °C to room temperature. Once complete, the reaction mixture was treated with saturated Na_2_CO_3_ solution and the compound was extracted with DCM (3 × 10 mL). The combined organic layers were rinsed with brine and dried over anhydrous MgSO_4_. The solvent was removed under reduced vacuum to reveal a bright orange oil. The oil was purified by flash chromatography on silica gel with the solvent system: 55/40/5 hexanes/dichloromethane/triethylamine. Bright orange oil, 49% yield. The free base was then converted to a phosphate salt with phosphoric acid.

**Yield:** 49%. **^1^H NMR** (400 MHz, deuterium oxide-*d*_2_) δ 9.26 (s, 1H), 8.26 (d, *J* = 9.0 Hz, 1H), 7.78 (d, *J* = 2.1 Hz, 1H), 7.69 (dd, *J* = 8.9, 2.2 Hz, 1H), 5.43 (p, *J* = 6.6 Hz, 1H), 3.19–3.09 (m, 6H), 2.47–2.34 (m, 1H), 2.28–2.15 (m, 1H), 1.81 (d, *J* = 6.6 Hz, 3H), 1.79–1.63 (m, 2H), 1.22–1.18 (m, 6H). **^13^C NMR** (126 MHz, deuterium oxide-*d*_2_) δ 144.70, 142.82, 139.06, 135.94, 133.40, 129.23, 126.81, 123.73, 113.20, 57.99, 50.95, 47.23, 32.16, 19.96, 19.89, 8.07 (d, *J* = 4.6 Hz). **^31^P NMR** (162 MHz, deuterium oxide-*d*_2_) δ 0.03. **HRMS** (ESI) calcd. for C_18_H_25_ClN_5_ [M + H]: 346.1798, found: 346.1793.


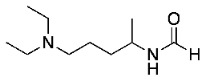
*N*-(5-(diethylamino)pentan-2-yl)formamide (**9**)

A mixture of 2-Amino-5-diethylaminopentane (5 g, 31.6 mmol) and ethyl formate (7.7 mL, 3 equiv.) was heated at 70 °C for 18 h in a round-bottom flask with molecular sieves. After completion, the mixture was cooled to room temperature and ethyl acetate was added. The resulting solution was washed with 1 M HCl and extracted with ethyl acetate (3 × 150 mL). The organic layer was then basified with 1 M NaOH and extracted with ethyl acetate (3 × 200 mL), and then rinsed with brine and dried over anhydrous magnesium sulfate. The solvent was evaporated under reduced pressure to obtain the *N*-(5-(diethylamino)pentan-2-yl)formamide product. Used without further purification. Orange oil, 5.689 g, 97% yield.

**Yield:** 97%. **Major trans rotamer**: **^1^H NMR** (500 MHz, chloroform-*d*) δ 8.10 (s, 1H), 6.55 (s, 1H), 4.08–3.97 (m, 1H), 2.53 (dd, *J* = 7.2, 4.7 Hz, 4H), 2.45–2.35 (m, 2H), 1.50 (dd, *J* = 6.2, 3.8 Hz, 4H), 1.15 (d, *J* = 6.6 Hz, 3H), 1.04–1.00 (m, 6H). **^13^C NMR** (126 MHz, chloroform-*d*) δ 160.68, 53.02, 46.77, 44.01, 34.80, 23.14, 20.84, 11.42. **Minor cis rotamer: ^1^H NMR** (500 MHz, chloroform-*d*) δ 8.07 (d, *J* = 12.0 Hz, 1H), 5.93 (s, 1H), 3.49 (s, 1H), 2.53 (dd, *J* = 7.2, 4.7 Hz, 4H), 2.45–2.35 (m, 2H), 1.50 (dd, *J* = 6.2, 3.8 Hz, 4H), 1.21 (d, *J* = 6.6 Hz, 3H), 0.99 (s, 6H). **^13^C NMR** (126 MHz, chloroform-*d*) δ 163.78, 52.65, 48.37, 46.86, 36.00, 23.58, 22.79, 11.62. **HRMS** (APCI) calcd. for C_10_H_23_ON_2_ [M + H]: 187.1804, found: 187.1803.


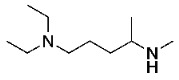
*N*^1^,*N*^1^-diethyl-*N*^4^-methylpentane-1,4-diamine (**10**)

To a suspension of 2 M LiAlH4 in THF, *N*-(5-(diethylamino)pentan-2-yl)formamide (4.5 g, 24.2 mmol) which was dissolved and degassed in anhydrous THF, was added portion-wise under stirring and cooling in an ice bath. The mixture was stirred at 0 °C for 30 min then the reaction mixture was refluxed for 24 h under nitrogen. After completion 1 M NaOH was added dropwise to hydrolyze any remaining LiAlH_4_. All precipitates were filtered off and washed with THF. The filtrate was then placed in a separatory funnel and the organic layer was separated. The organic layer was then acidified with 1 M HCl and extracted with dichloromethane (3 × 200 mL). The combined organic layers were then basified with sat. NaOH and extracted with dichloromethane (3 × 300 mL). The organic layers were then rinsed with brine and dried over anhydrous MgSO_4_, and the solvent was removed under reduced pressure to reveal pure *N*^1^,*N*^1^-diethyl-*N*^4^-methylpentane-1,4-diamine, which was used without further purification. Orange oil, 2.685 g, 65% yield.

**Yield:** 65% yield. **^1^H NMR** (500 MHz, chloroform-*d*) δ 2.52 (q, *J* = 7.2 Hz, 4H), 2.40 (d, *J* = 6.5 Hz, 5H), 1.51–1.42 (m, 3H), 1.34–1.23 (m, 1H), 1.05 (d, *J* = 6.3 Hz, 3H), 1.01 (t, *J* = 7.2 Hz, 6H). **^13^C NMR** (126 MHz, chloroform-*d*) δ 55.07, 53.32, 46.87, 34.91, 33.79, 23.68, 19.79, 11. **HRMS** (APCI) calcd. for C_10_H_25_N_2_ [M + H]: 173.2012, found: 173.2009.


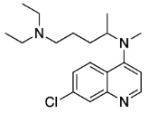
*N*4-(7-chloroquinolin-4-yl)-*N*1,*N*1-diethyl-*N*4-methylpentane-1,4-diamine (**6**)

The mixture of 4,7-dichloroquinoline (500 mg, 2.5 mmol) and *N*^1^,*N*^1^-diethyl-*N*^4^-methylpentane-1,4-diamine (652.6 mg, 3.8 mmol, 1.5 equiv.) were placed in a microwave flask with p-toluenesulfonic acid (816 mg, 4.3 mmol, 1.7 equiv.). This mixture was heated at 120 °C for 1.5 h. Once complete, the reaction was cooled to room temperature. The mixture was poured into ice water, and then 1 M NaOH was added. This was extracted with ethyl acetate (3 × 100 mL), washed with brine, and the organic layers were then dried over anhydrous MgSO_4_. The solvent was removed under reduced vacuum to reveal an orange oil. The oil was purified by flash chromatography on silica gel with solvent system: 50% hexanes, 50% ethyl acetate to 100% ethyl acetate and 5–10% triethylamine to give an orange oil 534.5 mg, 63% yield.

**Yield:** 63%. **^1^H NMR** (500 MHz, chloroform-*d*) δ 8.60 (d, *J* = 5.2 Hz, 1H), 7.99 (d, *J* = 2.2 Hz, 1H), 7.88 (d, *J* = 9.0 Hz, 1H), 7.35 (dd, *J* = 9.0, 2.2 Hz, 1H), 6.75 (d, *J* = 5.2 Hz, 1H), 3.85 (h, *J* = 6.8 Hz, 1H), 2.85 (s, 3H), 2.43 (qd, *J* = 7.1, 1.6 Hz, 4H), 2.34–2.29 (m, 2H), 1.76–1.65 (m, 1H), 1.56–1.47 (m, 1H), 1.46–1.31 (m, 0H), 1.25 (d, *J* = 6.5 Hz, 3H), 0.94 (t, *J* = 7.1 Hz, 6H). **^13^C NMR** (126 MHz, chloroform-*d*) δ 157.55, 151.53, 150.78, 134.64, 128.90, 125.98, 125.22, 121.86, 109.02, 58.80, 52.87, 46.91, 32.49, 32.35, 24.64, 17.10, 11.70. **HRMS** (APCI) calcd. for C_19_H_29_ClN_3_ [M + H^+^]: 334.2045, found: 334.2046.


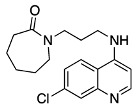
1-(3-((7-chloroquinolin-4-yl)amino)propyl)azepan-2-one

4,7-dichloroquinoline (126 mg, 0.64 mmol), *N*^1^,*N*^1^-diethyl-*N*4-methylpentane-1,4-diamine (100 mg, 0.58 mmol) and DBU (0.43 mL, 2.9 mmol) were placed in a round-bottom flask and heated to 125 °C. The mixture was stirred for 18 h. The brown mixture was then cooled to room temperature and washed with sat. NaOH and extracted with ethyl acetate (3 × 100 mL). The combined organic layers were then rinsed with water and dried over anhydrous MgSO_4_, and the solvent was removed under reduced pressure. The oil was purified by flash chromatography on silica gel with the solvent system: 85% ethyl acetate, 10% methanol, and 5% triethylamine to give a white solid, 82% yield.

**Yield:** 82%. **^1^H NMR** (500 MHz, chloroform-*d*) δ 8.46 (d, *J* = 5.5 Hz, 1H), 8.00 (d, *J* = 9.0 Hz, 1H), 7.95 (d, *J* = 2.2 Hz, 1H), 7.39 (dd, *J* = 9.0, 2.2 Hz, 1H), 7.02 (s, 1H), 6.39 (d, *J* = 5.6 Hz, 1H), 3.53–3.47 (m, 2H), 3.41–3.33 (m, 4H), 2.64–2.58 (m, 2H), 1.85–1.70 (m, 6H), 1.67 (p, *J* = 5.6 Hz, 2H). **^13^C NMR** (126 MHz, chloroform-*d*) δ 177.42, 151.23, 150.33, 148.73, 135.16, 127.91, 125.47, 122.25, 117.65, 98.09, 49.88, 45.11, 38.47, 37.17, 29.93, 28.44, 25.63, 23.46. **HRMS** (ESI) calcd. for C_18_H_23_ClN_3_O [M + H]: 332.1524, found: 332.1520.


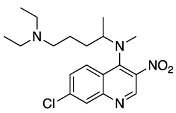
*N*4-(7-chloro-3-nitroquinolin-4-yl)-*N*1,*N*1-diethyl-*N*4-methylpentane-1,4-diamine (**16**)

To a solution of 4,7-dichloro-3-nitroquinoline (500 mg, 2.1 mmol) in acetonitrile was added *N*^1^,*N*^1^-diethyl-*N*^4^-methylpentane-1,4-diamine (709 mg, 4.1 mmol, 2 equiv.) and *N*,*N*-diisopropylethylamine (0.54 mL, 1.5 equiv.), which was stirred until it formed a homogenous solution. This mixture was stirred at room temperature for up to 10 min. The reaction mixture was then transferred to an 85 °C oil bath and stirred for 30 min. Once complete, the reaction mixture was evaporated to dryness under reduced pressure. The residue was diluted with ethyl acetate and water and basified with 1 M NaOH. The resulting solution was extracted with ethyl acetate (3 × 100 mL). The combined organic layers were then rinsed with brine and dried over anhydrous MgSO_4_. The solvent was removed under reduced pressure. The crude product was purified by flash silica column chromatography with 85/10/5 hexanes/ethyl acetate/triethylamine to reveal a bright orange solid, 719.7 mg, 92%.

**Yield:** 92%. **^1^H NMR** (500 MHz, chloroform-*d*) δ 8.93 (s, 1H), 8.06 (d, *J* = 2.2 Hz, 1H), 8.02 (d, *J* = 9.2 Hz, 1H), 7.50 (dd, *J* = 9.1, 2.2 Hz, 1H), 4.03–3.95 (m, 1H), 2.85 (s, 3H), 2.51 (d, *J* = 7.3 Hz, 4H), 2.42 (d, *J* = 7.7 Hz, 2H), 1.89–1.81 (m, 1H), 1.65 (dq, *J* = 13.5, 8.0 Hz, 1H), 1.47 (q, *J* = 7.8 Hz, 2H), 1.43 (d, *J* = 6.6 Hz, 3H), 1.00 (t, *J* = 7.1 Hz, 6H). **^13^C NMR** (126 MHz, chloroform-*d*) δ 151.13, 150.70, 147.90, 137.63, 137.04, 129.68, 127.55, 127.18, 123.82, 60.99, 52.76, 46.94, 33.91, 32.73, 24.46, 18.19, 11.42. **HRMS** (APCI) calcd. for C_19_H_27_ClN_4_O_2_ [M − H^+^]: 378.1828, found: 378.1822.


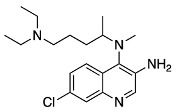
7-chloro-*N*4-(5-(diethylamino)pentan-2-yl)-*N*4-methylquinoline-3,4-diamine (**17**)

*N*4-(7-chloro-3-nitroquinolin-4-yl)-*N*1,*N*1-diethyl-*N*4-methylpentane-1,4-diamine (500 mg, 1.3 mmol) and stannous chloride (1.49 g, 5 equiv.) were placed in a round-bottom flask. Ethanol was added and the mixture was stirred at 80 °C for 2 h. Once complete, the mixture was cooled to room temperature. Once cool, the solvent was removed by reduced pressure. The residue was diluted with ethyl acetate and basified with sat NaOH and extracted with ethyl acetate (3 × 50 mL). The combined organic layers were washed with brine and dried over anhydrous MgSO_4_. The solvent was removed under reduced pressure to reveal a dark orange oil. This was purified by column chromatography with the solvent system: ethyl acetate (100–90%)/MeOH (0–10%)/NEt_3_ (5%) to give an orange oil, 313 mg, 68% yield.

**Yield:** 68%. **^1^H NMR** (500 MHz, dimethyl sulfoxide-*d*_6_) δ 8.53 (s, 1H), 7.88 (d, *J* = 9.0 Hz, 1H), 7.80 (d, *J* = 2.2 Hz, 1H), 7.40 (dd, *J* = 9.0, 2.3 Hz, 1H), 5.34 (s, 2H), 3.36 (q, *J* = 6.5, 5.5 Hz, 1H), 2.81 (s, 3H), 2.35 (q, *J* = 7.1 Hz, 4H), 2.25 (q, *J* = 5.9 Hz, 2H), 1.54 (d, *J* = 12.7 Hz, 1H), 1.44–1.29 (m, 2H), 1.03 (d, *J* = 6.4 Hz, 3H), 0.85 (t, *J* = 7.1 Hz, 7H). **HRMS** (ESI) calcd. for C_19_H_30_ClN_4_O_2_ [M + H^−^]: 349.2153, found: 349.2143.


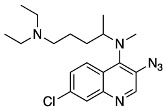
*N*4-(3-azido-7-chloroquinolin-4-yl)-*N*1,*N*1-diethyl-*N*4-methylpentane-1,4-diamine (**18**)

7-chloro-*N*4-(5-(diethylamino)pentan-2-yl)-*N*4-methylquinoline-3,4-diamine (330 mg, 0.95 mmol) was dissolved in a solution of concentrated sulfuric acid 98% and water (3.2 M). The resultant solution was cooled to 0 °C, and to this a solution of sodium nitrite in water (2.5 M) was added dropwise with stirring. The solution was stirred at this temperature for 5 min. Then, a solution of sodium azide in water (4.5 M) was added to it with vigorous stirring. The mixture was stirred for 5 min at 0 °C. The reaction mixture was then quenched with sat. NaCO_3_ and extracted with ethyl acetate (3 × 25 mL). The combined extracts were rinsed with brine and dried over anhydrous MgSO_4_, and the solvent was removed under reduced pressure to reveal a dark orange oil. This oil was purified by column chromatography with the solvent system: 65/35/5 ethyl acetate/hexanes/triethylamine to reveal a dark orange oil, 101 mg, 28%.

**Yield:** 28%. **^1^H NMR** (500 MHz, chloroform-*d*) δ 8.67 (s, 1H), 8.00 (d, *J* = 2.2 Hz, 1H), 7.98 (d, *J* = 9.0 Hz, 1H), 7.42 (dd, *J* = 9.0, 2.2 Hz, 1H), 3.49 (q, *J* = 6.4 Hz, 1H), 2.49 (q, *J* = 7.2 Hz, 4H), 2.38 (t, *J* = 7.3 Hz, 2H), 1.69–1.61 (m, 1H), 1.53–1.43 (m, 3H), 1.21 (d, *J* = 6.5 Hz, 3H), 0.98 (t, *J* = 7.1 Hz, 6H). **^13^C NMR** (126 MHz, chloroform-*d*) δ 147.93, 146.13, 145.08, 134.30, 128.91, 128.14, 127.48, 125.96, 125.91, 58.61, 53.02, 46.94, 35.52, 32.74, 24.11, 17.90, 11.54. **IR** (cm^−1^) 2966.6, 2933.2, 2110.6, 1559.3, 1451.4, 1381.8, 1319.9, 1073.4, 915.8. **HRMS** (ESI) calcd. for C_19_H_28_ClN_6_ [M + H^+^]: 375.2072, found: 375.2074.


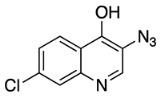
3-azido-7-chloroquinolin-4-ol (**19**)

See procedure for **17**.

Yield: 46%. ^1^H NMR (500 MHz, dimethyl sulfoxide-*d*_6_) δ 12.23 (d, *J* = 6.2 Hz, 1H), 8.11 (d, *J* = 8.7 Hz, 1H), 7.95 (d, *J* = 6.3 Hz, 1H), 7.63 (d, *J* = 2.0 Hz, 1H), 7.38 (dd, *J* = 8.7, 2.0 Hz, 1H). ^13^C NMR (126 MHz, dimethyl sulfoxide-*d*_6_) δ 171.72, 139.35, 136.45, 130.19, 127.04, 124.10, 122.37, 120.30, 117.71. IR (cm^−1^) 3075.6, 2124.7, 1627.9, 1558.2, 1512.3, 1460.4, 1350.8, 1188.7, 1075.2, 812.8. HRMS (ESI) calcd for C_9_H_5_ClN_4_O [M + Na^+^]: 243.0044, found: 243.0043.

### 6.3. X-ray Crystallography

Crystals for the ring-opened DBU adduct 1-(3-((7-chloroquinolin-4-yl)amino)propyl)azepan-2-one were grown from dichloromethane and hexanes. A large 0.1 mm × 0.1 mm × 0.06 mm prism was mounted on a glass fiber with epoxy resin. Single-crystal X-ray diffraction was then measured at room temperature with a BRUKER SMART CCD or BRUKER APEX-II CCD diffractometer by using graphite-monochromated Mo_Kα_ radiation (λ = 0.71073 Å). The crystal was crystallized in the monoclinic space group *P2_1_/c* with the unit cell parameters *a* = 10.65(2); *b* = 16.68(3); *c* = 10.68(2) Å and α = γ = 90 and β = 116.34(3)°, which corresponds to *V* = 1700.0(6) Å^3^ and *Z* = 4. SAINT [45] was used for integration of the intensity reflections and scaling and SADABS [45] for absorption correction. A combination of intrinsic phasing and direct methods were used for solving the structure, which were subsequently anisotropically refined after all non-hydrogen atoms were located by difference Fourier maps, and final solution refinements were solved by full-matrix least-squares method on *F*^2^ of all data by using SHELXTL (20141/7) [45] software. The hydrogen atoms were placed in calculated positions. The final refinement has *R_1_ =* 0.0340 and S*_gof_ =* 1.167. The full cif file has been submitted to the CCDC and is available with the code number 2312790.

### 6.4. UV–Vis Studies

First, 2.4 mg of 3-N_3_MeCQ was dissolved in 30 mL methanol to make a 213.4 μM stock solution. Then, 2.9 mg of 3-N_3_MeCQ was dissolved in 1 mL MeOH, and the resulting mixture was diluted with 29 mL H_2_O to make a 257.8 μM stock solution. Then, 3.8 mg of GlyGlyGly was dissolved in 30 mL deionized water to make a 669.6 μM stock solution. Finally, 6.5 mg of N-phenylmaleimide was dissolved in 30 mL methanol to make a 1251.2 μM stock solution.

### 6.5. General Procedure

A 2 mL blank solution of deionized water or methanol in a 4 mL quartz cuvette was taken in an Agilent UV–Vis Spectrophotometer for the area 190 nm–1100 nm. To a different 4 mL quartz cuvette was added 333 μL of the 257.8 μM stock solution and 1667 μL of deionized water to make a 42.97 μM 3-N_3_MeCQ solution. This was thoroughly mixed using a micropipette to make a homogeneous solution. A stir bar was placed in the cuvette, the cuvette was capped and placed in a cuvette holder fitted with a 365 nm pen light, and the setup was then covered with aluminum foil. The control spectrum was measured with the light off. The experiment was then started in kinetics mode for a spectrum to be taken every 10 s for 300 s whilst the light was on and the solution was stirring. When the experiment was finished, the resulting solution was transferred into a 2 mL Eppendorf tube for mass spectroscopy.

To a different 4 mL quartz cuvette, the stock 3-N_3_MeCQ solution was diluted with deionized water or methanol to make the desired concentrated solution. This was thoroughly mixed using a micropipette to make a homogeneous solution. A stir bar was placed in the cuvette, the cuvette was capped and placed in a cuvette holder fitted with a 365 nm pen light, and the setup was then covered with aluminum foil. The control spectrum was measured with the light off. The experiment was then started in kinetics mode for a spectrum to be taken every 10 s for 300 s whilst the light was on and the solution was stirring. When the experiment was finished, the resulting solution was transferred into a 2 mL Eppendorf tube for mass spectroscopy.

### 6.6. Insertion Experiments

First, 21.49 μM 3-N_3_MeCQ and GlyGlyGly in H_2_O (1:1); 5000 μL of the 257.8 μM 3-N_3_MeCQ stock solution was further diluted into 30 mL deionized water to make a 42.97 μM solution, and 1000 μL of this solution was then transferred into a 4 mL quartz cuvette. Then, 2000 μL of the 669.6 μM GlyGlyGly stock solution was further diluted into 30 mL deionized water to make a 44.64 μM, and 1000 μL of this solution was added into the above 4 mL cuvette to give final concentrations of 21.49 μM and 22.32 μM of 3-N_3_MeCQ and GlyGlyGly, respectively. The kinetics experiment was run as mentioned above.

For 4.29 μM 3-N_3_MeCQ and GlyGlyGly in H_2_O (1:9.4), 200 μL of the freshly made 42.97 μM 3-N_3_MeCQ solution was transferred into a 4 mL quartz cuvette. To this was added 1800 μL of the freshly made 44.64 μM GlyGlyGly solution to give final concentrations of 4.29 μM and 40.18 μM of 3-N_3_MeCQ and GlyGlyGly, respectively. The kinetics experiment was run as mentioned above.

For 19.56 μM 3-N_3_MeCQ and N-phenylmaleimide in MeOH (1:1), 5000 μL of the 213.4 μM 3-N_3_MeCQ stock solution was further diluted into 30 mL methanol to make a 35.57 μM solution, and 1100 μL of this solution was then transferred into a 4 mL quartz cuvette. Then, 1000 μL of the 1251.2 μM N-phenylmaleimide stock solution was further diluted into 30 mL methanol to make a 41.71 μM solution, and 900 μL of this solution was added into the above 4 mL cuvette to give final concentrations of 19.56 μM and 18.78 μM of 3-N_3_MeCQ and N-phenylmaleimide, respectively. The kinetics experiment was run as mentioned above.

For 7.11 μM 3-N_3_MeCQ and N-phenylmaleimide in MeOH (1:4.7), 400 μL of the freshly made 35.57 μM 3-N_3_MeCQ solution was transferred into a 4 mL quartz cuvette. To this was added 1600 μL of the freshly made 41.71 μM N-phenylmaleimide solution to give final concentrations of 7.11 μM and 33.37 μM of 3-N_3_MeCQ and N-phenylmaleimide, respectively. The kinetics experiment was run as mentioned above.

3.56 μM 3-N_3_MeCQ and N-phenylmaleimide in MeOH (1:10.6), 200 μL of the freshly made 35.57 μM 3-N_3_MeCQ solution was transferred into a 4 mL quartz cuvette. To this was added 1800 μL of the freshly made 41.71 μM N-phenylmaleimide solution to give final concentrations of 3.56 μM and 37.54 μM of 3-N_3_MeCQ and N-phenylmaleimide, respectively. The kinetics experiment was run as mentioned above.

### 6.7. NMR Experiments

To a 0.5 dram vial was added 3-N_3_MeCQ, the electron acceptor, and 500 μL of deuterated methanol. This was thoroughly mixed to make a homogenous solution. This mixture was then transferred into an 8″ NMR tube fitted with a stir bar. A 365 nm pen light was positioned 5 cm from the NMR tube. In a dark fume hood with the setup covered in aluminum foil, the solution was stirred exposed to 365 nm for 6 to 9 min. Once the time elapsed, the stir bar was removed and a ^1^H NMR spectrum was measured.

For 3-N_3_MeCQ and N-phenylmaleimide, 5 mg of 3-N_3_MeCQ and 2.3 mg of N-Phenylmaleimide were used and irradiated for 6 min.

For N-phenylmaleimide, 2.3 mg of N-Phenylmaleimide used and irradiated for 9 min. For 3-N_3_MeCQ and tetracyanoethylene, 5 mg of 3-N_3_MeCQ and 2.3 mg of tetracyanoethylene were used and irradiated for 9 min.

## Figures and Tables

**Table 1:**
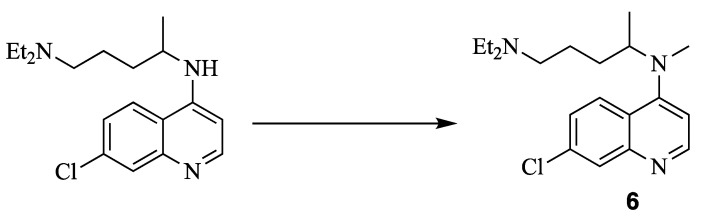
Methylation of chloroquine with methyl iodide.

Base	Solvent	Methylating Agent	Temperature (°C)	Yield (%)
NaH	DMF	MeI	−20 to 0	23
NaH	DMF	MeI	Room temp.	15
NaH	DMF	MeI	−20	9
NaH	DMF	MeI	−20	N.D.
NaH	MeCN	MeI	−20	-

**Table 2:**
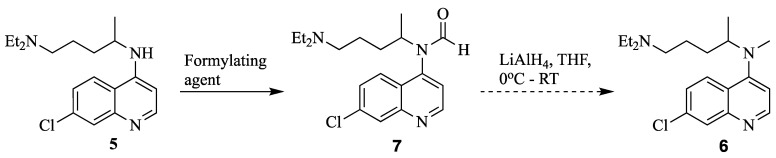
Attempted methylation of chloroquine with formic acid and sodium ethoxide.

Formylating Agent	Solvent	Temperature (°C)	Catalyst	Yield (%)
Formic acid	-	70–84	-	-
Formic acid	-	70	I_2_	-
Formamide	THF	RT–70	NaOMe	-
Sodium ethoxide	CHCl_3_	55	-	-

**Table 3:**
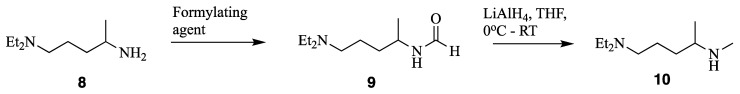
Methylation of 2-amino-5-diethylaminopentane with formic acid and ethyl formate.

Formylating Agent	Solvent	Temperature (°C)	Yield (%)
Formic acid	-	100	57
Formic acid, acetic anhydride	-	60	-
Ethyl formate	-	70	97

**Table 4:**
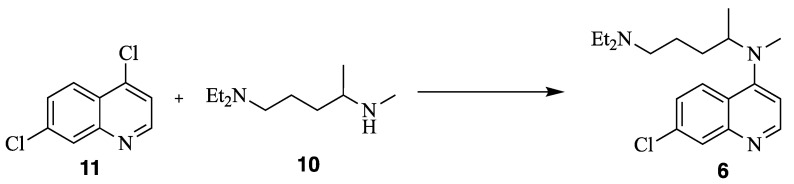
Condensation reaction between DCQ and methylated side chain under basic conditions.

Base	Solvent	Temperature (°C)	Yield (%)
**-**	-	85	-
**-**	-	120–125	6
**-**	-	120 ^a^	-
**-**	Ethylene glycol	120 ^a^	6
**NEt_3_**	-	125	-
**NEt_3_**	-	85 ^a^	-
**NEt_3_**	EtOH	0–40	-
**DBU**	-	80 ^a^	10
**DBU**	-	125	82 ^b^
**DIPEA**	DMSO	80	-
**DIPEA**	DMSO	120	-
**DIPEA**	MeCN	85	-
**^t^** **BuOK**	DMSO	80–120	Decomp.
**^t^** **BuOK**	THF	RT–80 °C	-
**^c^**	1,4-dioxane	85	8

^a^ Microwave-assisted reaction, ^b^ yield of side product, ^c^ Pd(OAc)_2_, DPEPhos, K_3_PO.

**Table 5:** Condensation reactions conditions for 7-chloroquinolin-4-yl trifluoromethanesulfonate and the methylated side chain.

Base	Solvent	Temperature (°C)	Yield (%)
-	-	80–125	24
DIPEA	DMSO	80–125	trace
DIPEA	Acetonitrile	RT–40	15
DIPEA	-	125	26
NEt_3_	-	80–125	22
NEt_3_	EtOH	0–40	-

**Table 6:** Condensation reaction between DCQ and methylated side chain under acidic conditions.

Acid	Solvent	Temperature (°C)	Yield (%)
Phenol ^a^	-	125	14 ^b^
Phenol	-	125 ^c^	16
p-toluenesulfonic acid	DMSO	125	-
p-toluenesulfonic acid	-	120 ^c^	63

^a^ Reaction mixture includes 1.2 eq NEt_3_. ^b^ 36% of 7-chloro-4-phenoxyquinoline formed. ^c^ microwave-assisted reaction.

## Data Availability

Data are contained within the article.
